# Exploring the role and therapeutic potential of RNA N6-methyladenosine modification in abortion disease pathology: a comprehensive review

**DOI:** 10.3389/fgene.2025.1720842

**Published:** 2026-01-07

**Authors:** Zhuo Chang, Lu-Hao Li, Liang-Zhen Lv, Zhao-Di Wang, Qing-yi Wang, Hui Zhu, Bei Jiang, Xue-Ming Zhou, Ya-Peng Han, Xue Pan, Li Ren, Sen Cheng, Zi-Meng Lei

**Affiliations:** 1 Heilongjiang University of Chinese Medicine, Harbin, China; 2 The Third School of Clinical Medicine (School of Rehabilitation Medicine) of Zhejiang Chinese Medical University, Hangzhou, China; 3 Shanghai University of Traditional Chinese Medicine, Shanghai, China; 4 First Affiliated Hospital, Heilongjiang University of Chinese Medicine, Harbin, China; 5 Third Affiliated Hospital, Beijing University of Chinese Medicine, Beijing, China; 6 Shunde Women and Children’s Hospital of Guangdong Medical University, Shunde, China

**Keywords:** embryo, epigenetics, immune microenvironment, interface, m6A methylation, maternal-fetal, miscarriage, obesity

## Abstract

Recurrent spontaneous abortion (RSA), defined as two or more consecutive pregnancy losses, affects 1%–5% of couples and poses a significant challenge to reproductive health. Despite its prevalence, the underlying etiology remains elusive in approximately half of all cases, hindering the development of targeted therapies. The emerging field of epitranscriptomics, particularly the dynamic and reversible N6-methyladenosine (m6A) RNA modification, offers a novel lens through which to investigate the complex gene-environment interactions underlying RSA. This review systematically synthesizes current knowledge on the pivotal roles of m6A modification in key processes essential for a successful pregnancy: gametogenesis and early embryo quality, placental development and function, and the establishment of immune tolerance at the maternal-fetal interface. We critically evaluate the direct and indirect evidence linking dysregulation of specific m6A regulators to the pathophysiology of RSA, drawing from human tissue studies, RSA animal models, and insights extrapolated from related fields.Furthermore, we discuss the translational potential and considerable challenges of targeting the m6A machinery for therapeutic intervention in RSA. This review aims not only to summarize the current landscape but also to provide a critical framework to guide future mechanistic and clinical research in this promising area.

## 1 Background

Recurrent spontaneous abortion (RSA) is a devastating disorder, classically defined as three or more consecutive losses before 28 weeks (Practice Committee of the American Society for Reproductive, 2012), with modern guidelines recognizing two or more losses as clinically significant. It affects 1%–5% of couples and inflicts profound physical and psychological burdens (Rai and Regan, 2006), (Turesheva et al., 2023). Alarmingly, the pathogenesis remains unclear in over 60% of cases (Jaslow et al., 2010),creating a major barrier to effective treatment. This underscores the urgent need to explore novel molecular pathways governing pregnancy establishment and maintenance.

Successful pregnancy requires flawless execution of interconnected events: competent embryo development, implantation, placentation via trophoblast invasion and decidualization, and establishment of local immune tolerance. Epigenetic regulation, particularly RNA modification, has emerged as a key mechanism controlling these processes (Zhao et al., 2020; Sendinc and Shi, 2023). Among these,N6-methyladenosine (m6A)—the most abundant internal mRNA modification in eukaryotes—stands out for its dynamic and reversible nature, enabling precise post-transcriptional control of gene expression (Roundtree et al., 2017; Huang N. et al., 2022; Dominissini et al., 2012; Gilbert et al., 2016).

The m6A modification is installed by methyltransferase complexes (“writers” like METTL3/METTL14/WTAP), removed by demethylases (“erasers” like FTO/ALKBH5), and interpreted by binding proteins (“readers” like the YTHDF family) that direct RNA fate, influencing splicing, stability, and translation (Shi et al., 2019; Jia et al., 2011; Zhang et al., 2010; Wang and Lu, 2021; Shi et al., 2017).While m6A’s role in fields like oncology is well-established (Willyard, 2017; Lee et al., 2014), its importance in reproduction is increasingly recognized (Huang and Chen, 2023).It is integral to key reproductive events, from gametogenesis to placental development and immune regulation (Zhao et al., 2022).

However, research directly investigating m6A in RSA-specific pathogenesis remains nascent and fragmented. Insights are often extrapolated from other pregnancy complications or non-reproductive models. Therefore, this review aims to provide a comprehensive and critical analysis of m6A’s role in RSA. We will synthesize physiological functions, evaluate direct and indirect pathogenic evidence, identify conflicts and gaps, and discuss therapeutic potential and challenges.

### 1.1 The mechanisms and regulation of m6A RNA methylation

Epigenetics plays a pivotal role in regulating gene expression, which is crucial for both disease development and prevention. Among the known epigenetic mechanisms—including DNA modification, histone modification, RNA modification, and chromatin remodeling—RNA modification has become a major research focus (Zhao et al., 2020). RNA molecules undergo approximately 160 types of chemical modifications, with methylation being the most prevalent (Sendinc and Shi, 2023).

In eukaryotes, N6-methyladenosine (m6A) is the most abundant internal modification on messenger RNA (mRNA) and long non-coding RNAs, occurring at the sixth nitrogen position of adenosine (Roundtree et al., 2017; Huang N. et al., 2022; Dominissini et al., 2012). This modification is enriched near stop codons and within 3′untranslated regions. m6A dynamically influences multiple aspects of RNA metabolism, including splicing, translational efficiency, nuclear export, stability, and decay. Importantly, m6A modification is reversible, which underpins its significant regulatory potential and therapeutic prospects (Gilbert et al., 2016; Berulava et al., 2020).

**FIGURE 1:**
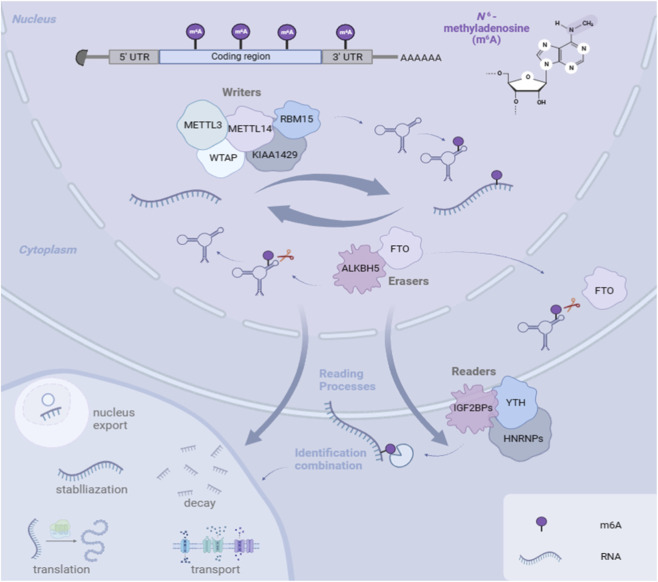
The mechanism of m6A readers, writers, and erasers.

The m6A modification process is regulated by three classes of proteins: methyltransferases (“writers”), demethylases (“erasers”), and binding proteins (“readers”) (Figure 1).

#### 1.1.1 Methyltransferases

Methyltransferases, or writers, catalyze the addition of a methyl group to adenosine on RNA. The core N6-adenosine methyltransferase complex (MTC) in mammals includes methyltransferase-like protein 3 (METTL3), methyltransferase-like protein 14 (METTL14), and Wilms’ tumor 1-associating protein (WTAP) (Roundtree et al., 2017). METTL3 is the catalytic core, directly binding to the methyl donor S-adenosylmethionine (Bokar et al., 1997; Xiong et al., 2022). METTL14, while lacking independent catalytic activity, forms a stable heterodimer with METTL3, enhancing RNA binding and complex stability (Wang et al., 2016; Lin et al., 2017). WTAP does not possess catalytic activity but is essential for localizing the METTL3-METTL14 complex to nuclear speckles, thereby guiding site-specific methylation (Huang Q. et al., 2022; Ping et al., 2014). Recent studies have identified additional regulatory components, such as RNA binding motif protein 15 (RBM15), Vir-like m6A methyltransferase associated (VIRMA/KIAA1429), and zinc finger CCCH domain-containing protein 13 (ZC3H13), which contribute to the specificity and efficiency of m6A deposition (Jia et al., 2021; Wang Y. et al., 2021; Wang et al., 2020).

#### 1.1.2 Demethylation enzymes

Demethylases, or erasers, remove m6A marks, making this modification reversible. The two primary m6A demethylases are fat mass and obesity-associated protein (FTO) and AlkB homolog 5 (ALKBH5), both belonging to the α-ketoglutarate-dependent dioxygenase family (Jia et al., 2022). The discovery of FTO’s demethylase activity in 2011 overturned the long-held view of static RNA modification, confirming the dynamic nature of m6A (Fu et al., 2013). FTO is distributed in both the nucleus and cytoplasm, whereas ALKBH5 is primarily nuclear. Both enzymes remove m6A through an Fe(II)- and α-ketoglutarate-dependent oxidative process (Aik et al., 2014).

#### 1.1.3 Binding proteins

Or readers, specifically recognize m6A-modified RNAs and mediate their functional outcomes. The YT521-B homology (YTH) domain family is a major class of readers, including cytoplasmic members (YTHDF1, YTHDF2, YTHDF3) and nuclear members (YTHDC1, YTHDC2) (Zhang et al., 2010). They perform distinct functions: YTHDF1 and YTHDF3 promote translation, while YTHDF2 facilitates mRNA decay (Wang and Lu, 2021; Shi et al., 2017). YTHDC1 regulates mRNA splicing and nuclear export, and YTHDC2 can promote translation while also affecting RNA stability (Xiao et al., 2016). Other important readers include insulin-like growth factor 2 mRNA-binding proteins (IGF2BPs), which stabilize target mRNAs (Huang et al., 2018; Duan et al., 2024), and heterogeneous nuclear ribonucleoproteins (HNRNPs), which are involved in splicing regulation (Zhou et al., 2019; Liu et al., 2015; Alarcon et al., 2015).

The coordinated actions of writers, erasers, and readers establish m6A as a precise and reversible layer of post-transcriptional regulation, influencing diverse biological processes and disease states.

### 1.2 Analyzing RSA pathogenesis from the perspective of the potential role of m6A methylation modification

A successful pregnancy involves establishing and maintaining pregnancy throughout its stages, culminating in labor. Failure can lead to adverse pregnancy outcomes. RSA, as an early pregnancy failure, has complex and multiple causes. Understanding it involves examining crucial stages of normal pregnancy maintenance, including normal embryogenesis, maternal-fetal interface establishment, and immune microenvironment regulation at this interface. These stages require several cytokines, each regulated at the RNA transcriptional level. Therefore, m6A methylation modifications affecting RNA transcription are crucial in gestation and influence pregnancy outcomes.

### 1.3 Biological functions and pathological effects of m6A methylation modifications in pregnancy establishment and RSA

#### 1.3.1 Embryogenesis and development

Embryogenesis and development are critical for establishing pregnancy, as embryo quality is a major determinant of early pregnancy outcome. This process encompasses oogenesis, spermatogenesis, fertilization, and subsequent embryonic cell proliferation and differentiation. Evidence indicates that m6A methylation regulates oogenesis, spermatogenesis, and embryonic development, thereby affecting the quality of the fertilized egg and the early embryo.

##### 1.3.1.1 Oogenesis

In mammals, the development of a mature, fertilization-competent oocyte follows a defined pathway: primordial germ cells differentiate into oogonia, which enter meiosis to form primary oocytes. These become enclosed by granulosa cells to form primordial follicles. After puberty, selected follicles mature and release a fertilizable oocyte. The quality of this process is paramount for female fertility and successful pregnancy (Laisk et al., 2019; Feng et al., 2014; Li and Albertini, 2013).

Previous studies have demonstrated that m6A methylation regulates various cellular processes in the female reproductive system, including cell proliferation, differentiation, metabolism, and the cell cycle (Huang and Chen, 2023). Direct evidence confirms its essential role in oogenesis. Reader proteins such as YTHDC1, YTHDC2, YTHDF1, and YTHDF2 participate in oocyte formation, development, and maturation by modulating RNA transcription and translation, which is critical for early embryogenesis (Kasowitz et al., 2018; Qi et al., 2022; Zhao et al., 2021; Ivanova et al., 2017). Nuclear YTHDC1 promotes pre-mRNA splicing; its deletion leads to oocyte arrest at the primary follicle stage and infertility (Kasowitz et al., 2018). Cytoplasmic YTHDF2 mediates maternal mRNA degradation during meiotic maturation, ensuring proper transcriptome construction in mature oocytes and facilitating early embryonic cleavage (Kasowitz et al., 2018; Zhao et al., 2021).

Methyltransferases (writers) such as METTL3 and METTL14 are also key for follicular development and oocyte maturation (Lin et al., 2017). Demethylases (erasers) such as ALKBH5 regulate maternal mRNA degradation by removing m6A marks. ALKBH5 deficiency leads to abnormal m6A accumulation, excessive translation, meiotic defects, chromosomal misalignment, spindle anomalies, and ultimately infertility (Bai et al., 2023). Another demethylase, FTO, maintains chromatin stability and accessibility by acting on long-interspersed element-1 (LINE1) RNA; loss of FTO causes LINE1 RNA degradation, chromatin compaction, and abnormal oocyte development (Wei et al., 2022).

Furthermore, differentially expressed genes involved in steroidogenesis, granulosa cell proliferation, and follicular development are highly enriched in m6A modifications, indicating that m6A not only regulates mRNA fate within oocytes but also indirectly influences the follicular microenvironment via granulosa cells (Cao et al., 2020). Dynamic changes in m6A modifications within granulosa cells across different follicular stages suggest involvement in regulating the oocyte-supporting environment (Cao et al., 2020).

Additionally, m6A modification is closely associated with long non-coding RNAs (lncRNAs) in reproductive disorders. lncRNAs are themselves highly enriched in m6A marks and play crucial roles in embryo implantation. In the endometrium of patients with recurrent implantation failure (RIF), m6A modification patterns of lncRNAs are significantly altered, affecting the expression of key implantation-related factors such as LIF, thereby disrupting the implantation window (Zhang L. et al., 2021; Liu HT. et al., 2022; Wang et al., 2024).

##### 1.3.1.2 Spermatogenesis

In mammals, spermatogenesis not only involves the stages of proliferation, meiosis, and spermiogenesis but is also critically regulated by epigenetic mechanisms, particularly N6-methyladenosine (m6A) modification (Tang et al., 2018). As the most prevalent modification of eukaryotic mRNA, m6A plays an essential role in maintaining the homeostasis, proliferation, and differentiation of spermatogonial stem cells (SSCs) into spermatocytes (Tang et al., 2018). The loss of m6A-regulating methyltransferases METTL3 and METTL14 disrupts post-transcriptional regulation in SSCs, leading to impaired spermatogenesis, indicating that m6A is deeply involved in the early stages of sperm development (Lin et al., 2017).

As SSCs transition to spermatocytes and enter meiosis, m6A reader proteins such as YTHDC2 begin to play a key role at the molecular level (Hsu et al., 2017). YTHDC2 regulates the translation and degradation of target mRNAs by binding to m6A-modified sites. Its expression is significantly upregulated prior to the onset of meiosis in spermatocytes, and its deletion results in meiotic arrest at the zygotene stage, blocking further spermatogenesis and ultimately inducing spermatocyte apoptosis (Hsu et al., 2017). Additionally, YTHDC2 interacts with multiple mRNA metabolic complexes, including XRN1, UPF1, and MOV10, to control transcript fate and maintain mRNA homeostasis during spermatogenesis (Hsu et al., 2017).

In the later stages of spermatogenesis, particularly during the haploid phase, the m6A demethylase ALKBH5 primarily functions in round spermatids (Tang et al., 2018). By removing m6A marks from long 3′-UTR transcripts, ALKBH5 ensures proper splicing and stable storage of these early-reserved mRNAs, thereby supporting late-stage sperm assembly (Tang et al., 2018). However, the loss of ALKBH5 function leads to aberrant splicing, with transcripts being incorrectly processed into truncated fragments and prematurely degraded. This results in disrupted expression of proteins essential for spermiogenesis and causes defective sperm development and infertility (Tang et al., 2018).

Even after the completion of spermatogenesis, the subsequent functional performance of sperm remains influenced by m6A regulation. Processes such as the acrosome reaction, capacitation, and fusion with the oocyte rely not only on structural maturation but also on the regulated expression of sperm-specific mRNAs and proteins. m6A-mediated regulation of mRNA stability and translational efficiency during these stages may indirectly affect sperm viability and fertilization potential.

Moreover, the regulatory role of m6A extends into early embryonic development (Wang et al., 2023). From the zygote to the blastocyst stage, m6A modification is crucial for maternal mRNA degradation, zygotic genome activation (ZGA), and embryonic fate determination. Studies have revealed that key developmental transcription factors such as Nanog, Sox2, and Oct4 carry m6A marks, which are closely associated with their mRNA stability and translational efficiency. In addition, m6A enhances miRNA-mediated degradation of maternal transcripts and regulates the expression of retrotransposons, playing a vital role in maintaining epigenetic homeostasis during embryogenesis (Wang et al., 2023).

Therefore, from SSC fate determination and the successful progression of meiosis to the maturation of haploid sperm and their roles in fertilization and embryonic development, m6A modifications and their regulatory factors—such as METTL3/METTL14, YTHDC2, and ALKBH5—form an epigenetic regulatory axis that spans the entire process of spermatogenesis and extends into early embryogenesis. This provides critical insights into the multilayered mechanisms underlying sperm development and the molecular basis of male infertility (Liu HT. et al., 2022; Wang et al., 2024).

#### 1.3.2 Establishment and development of the maternal-fetal interface

The maternal-fetal interface, located between the mother and the fetus, includes maternal meconium and fetal placenta components. This interface is crucial for maintaining pregnancy by providing nutritional support, acting as a protective barrier for the fetus, and ensuring maternal immune tolerance (Tang et al., 2018). Inadequate development of this interface can lead to adverse pregnancy outcomes, significantly RSA (Wang et al., 2023). Placental tissue predominantly contains trophoblast cells, while endometrial tissue comprises stromal cells undergoing endometrial metamorphosis. Recent studies increasingly show the significant role of RNA m6A modification in cell proliferation, differentiation, invasion, and endometrial metamorphosis, affecting the establishment and development of the maternal-fetal interface.

##### 1.3.2.1 Trophoblast invasion

During early implantation, trophoblast cells adhere to the maternal endometrium, proliferate, differentiate, and invade the decidua and spiral artery walls. In normal pregnancy, this invasion induces vascular remodeling to ensure adequate blood supply. In contrast, restricted trophoblast invasion is observed in conditions like early-onset preeclampsia (ePE) and intrauterine growth restriction (IUGR), leading to failed spiral artery remodeling and placental insufficiency (Kadyrov et al., 2006).

Aberrant invasion is closely associated with dysregulated expression of m6A-modifying enzymes in trophoblasts (Huang N. et al., 2022). For instance, WTAP expression is downregulated in ePE. Its loss reduces m6A methylation on downstream target HMGN3 mRNA, compromising its stability and weakening its promotive effect on trophoblast migration and invasion. This effect is mediated through reader protein IGF2BP1 (Bian et al., 2022).

In RSA villous tissues, the core writer METTL3 is significantly downregulated. One of its targets, ZBTB4—a transcription factor that inhibits migration and proliferation—exhibits increased mRNA stability and expression when m6A is reduced, thereby suppressing trophoblast invasion. METTL3-mediated m6A modification shortens ZBTB4 mRNA half-life; METTL3 insufficiency allows ZBTB4 to escape degradation (Huang N. et al., 2022).

Conversely, the demethylase ALKBH5 is abnormally upregulated in RSA. High ALKBH5 expression reduces the m6A level and stability of target gene CYR61 (an extracellular matrix protein critical for placentation), weakening its supportive function in trophoblast migration and invasion (Li et al., 2019). ALKBH5 overexpression is also associated with ferroptosis regulation, potentially influencing trophoblast survival by modulating genes like ferritin light chain (FTL) (Xie et al., 2016).

Excessive trophoblast apoptosis is another key factor in ePE and IUGR. Interestingly, in maternal anemia, trophoblast invasiveness is enhanced despite hypoxia, suggesting that apoptosis, rather than hypoxia *per se*, may be the primary mechanism affecting vascular remodeling in some pathologies (Kadyrov et al., 2006). m6A modification is involved in regulating programmed cell death pathways, including ferroptosis (Yang and Stockwell, 2016). ALKBH5 expression changes may influence FTL expression, modulating cellular iron storage and trophoblast sensitivity to ferroptosis (Gentric et al., 2019).

Furthermore, circular RNAs (circRNAs) are extensively involved in trophoblast function. Multiple circRNA–miRNA–mRNA axes have been confirmed in pregnancy complications. For example, circ_0017068 induces M1 macrophage polarization and trophoblast apoptosis via the miR-512-5p/MITA pathway and is implicated in RSA (Liu et al., 2023). Others like circ_0074371, circ_0011460, and circ_0037078 play roles in fetal growth restriction and preeclampsia by regulating trophoblast behavior (Zhou et al., 2018; Fan et al., 2021; Yao et al., 2022; Zou and Mao, 2022).

##### 1.3.2.2 Decidualization

Before embryo implantation, the endometrium must undergo decidualization, a process where endometrial stromal cells (ESCs) transform into decidual stromal cells (DSCs) under specific hormonal cues (Kagawa et al., 2022; Lee and DeMayo, 2004; Gellersen and Brosens, 2014; Owusu-Akyaw et al., 2019). Impaired decidualization can lead to reduced uterine receptivity, inadequate vascular remodeling, and insufficient fetal nutrient supply, all contributing to recurrent miscarriage (Ng et al., 2020; Salker et al., 2010; Lucas et al., 2020; Chang et al., 2022).

Various m6A-regulating enzymes modulate decidualization. METTL3, as an m6A writer, suppresses the expression of key decidualization genes such as progesterone receptor (PGR) (Zheng et al., 2023), forkhead box O1 (FOXO1) (Li et al., 2023), and homeobox A10 (HOXA10) (Xue et al., 2021) through m6A-mediated mRNA degradation. METTL3-mediated m6A enhances the binding of reader YTHDF2 to target mRNAs, promoting their decay and weakening DSC functional transformation (Li et al., 2023). Increased enhancer of zeste homolog 2 (EZH2) expression in ESCs from endometriosis patients is linked to elevated m6A levels and repression of decidual genes like IGFBP1, suggesting crosstalk between m6A and histone modifications (Lin et al., 2024).

A clear connection exists among m6A imbalance, decidualization defects, and miscarriage. Biological samples from miscarriage patients show decreased FTO and increased METTL3 expression, accompanied by downregulation of decidual markers (IGFBP1,PRL,FOXO1), supporting that m6A imbalance disrupts DSC formation (Qiu et al., 2021). FTO downregulation increases m6A burden on key mRNAs (e.g.,,HLA-G,VEGFR), affecting immune tolerance and angiogenesis at the maternal-fetal interface (Qiu et al., 2021). The reader YTHDF2 regulates EZH2 mRNA stability, epigenetically suppressing decidualization-related genes and aggravating decidual defects (Lin et al., 2024).

CircRNA expression is also abnormal in decidua of patients with pathological pregnancy loss and is closely linked to m6A modifications. Certain circRNAs may regulate m6A modulator expression via competing endogenous RNA (ceRNA) mechanisms, indirectly influencing methylation levels of key decidual mRNAs (Cui et al., 2023).

#### 1.3.3 Immune microenvironment regulation at the maternal-fetal interface

The maintenance of a successful pregnancy relies on a specialized immune microenvironment that facilitates maternal immune tolerance toward fetal alloantigens, preventing immunological conflict. A substantial body of research has established a close association between immune microenvironment imbalance and recurrent spontaneous abortion (RSA), particularly in unexplained cases where immune rejection is often a primary cause (Recurrent abortion, 1993; Alijotas-Reig et al., 2014; Zenclussen et al., 2007; La Rocca et al., 2014). This microenvironment comprises various immune cells, including decidual natural killer (dNK) cells, decidual macrophages (dMΦ), and decidual T cells, which collectively maintain a dynamic equilibrium balancing tolerance and defense (Xu et al., 2021).

In normal pregnancy, dNK cells exhibit a low-cytotoxicity, high-secretory phenotype (CD56^∧^brightCD16^∧^-), assisting in spiral artery remodeling and receiving inhibitory signals from fetal HLA molecules to maintain tolerance (Xu et al., 2021). dMΦ are predominantly polarized toward the M2 phenotype, contributing to angiogenesis and tissue repair (Erlebacher, 2013). Regulatory T cells (Tregs) migrate to the interface, suppressing effector T cell activity through cytokines like TGF-β and IL-10, thereby preventing fetal rejection (Alijotas-Reig et al., 2014).

As a post-transcriptional epigenetic regulator, RNA m6A methylation plays a crucial role in maintaining immune homeostasis at the maternal-fetal interface. “Writer” enzymes such as METTL3, METTL14, and WTAP add m6A modifications to specific transcripts, affecting the development and function of T cells, NK cells, and macrophages. For example, METTL3 can promote the degradation of SOCS family mRNAs in CD4^+^ T cells, activating the IL-7–STAT5 axis to maintain T cell homeostasis (Wu et al., 2023). In Tregs, m6A modification enhances their suppressive function (Wu et al., 2023). Conversely, “eraser” enzymes such as FTO and ALKBH5 remove m6A marks to modulate the expression of key factors like VEGF and STAT1, influencing macrophage polarization and NK cell function (Wu et al., 2023).

In RSA, immune microenvironment imbalance is closely linked to aberrant m6A modification patterns (Tsui et al., 2006). Inflammatory stimuli such as IL-6 and IL-1β can drive naïve CD4^+^ T cells toward pro-inflammatory Th17 differentiation while suppressing Treg formation, intensifying local inflammation and impairing implantation (Bansal, 2010). Studies have shown that uterine tissues from RSA patients exhibit decreased expression of METTL3 and WTAP, along with elevated expression of FTO (Tsui et al., 2006). These changes may interfere with T cell differentiation, decidualization, trophoblast invasion, and vascular remodeling, increasing the risk of pregnancy failure (Tsui et al., 2006).

Moreover, m6A modification is involved in regulating inflammatory responses. During pathogenic invasion or inflammation, METTL3/14 expression can be upregulated, increasing m6A methylation and activating pro-inflammatory pathways. Melatonin, through its receptor MTNR1B, may modulate this process by restoring FTO function and reducing METTL3 expression, thereby helping to maintain the immunosuppressive environment essential for early pregnancy (Zhao et al., 2024).

### 1.4 Correlation between m6A methylation modifications in risk factors and RSA occurrence

Successful pregnancy is a long-term and complex process with several risk factors that can lead to miscarriage. Addressing these risks at their source is crucial for treating recurrent miscarriages, making it a significant aspect of RSA exploration. Accepted factors, such as autoimmune diseases, obesity, and endocrine imbalances, are widely recognized. Recent studies have found the biological role of m6A methylation in these risk factors, offering new insights into RSA.

#### 1.4.1 Autoimmune dieases

The immune environment is critical for preventing maternal-fetal conflict. Dysregulated immune responses are closely associated with an increased risk of RSA (Alecsandru et al., 2021; Vomstein et al., 2021). The human leukocyte antigen (HLA) system, including allelic polymorphisms and maternal-fetal incompatibility, may contribute to RSA pathogenesis (Prins et al., 2014; Meuleman et al., 2015; Meuleman et al., 2018). Specific combinations of fetal HLA-C and maternal killer-cell immunoglobulin-like receptors (KIRs) can lead to either insufficient placental invasion or heightened immune aggression, triggering miscarriage (Vomstein et al., 2021). HLA class II genes (e.g.,,DRB1,DQB1) influence immune tolerance; susceptible alleles may enhance pro-inflammatory T cell activation and impair regulatory T cell (Treg) differentiation, increasing fetal rejection risk (Aimagambetova et al., 2019). Maternal immunological memory against fetal HY antigens (from a male fetus) can also contribute to miscarriage in subsequent pregnancies (Aruna et al., 2011).

The transcription factor FOXP3 is pivotal for Treg differentiation and function. Polymorphisms in the FOXP3 gene may affect its expression and influence RSA susceptibility (Abdukassimova et al., 2021). Importantly, m6A modification plays a key role in regulating FOXP3 expression by modulating mRNA stability and translational efficiency (Bahia et al., 2022). Specifically, METTL14-catalyzed m6A helps sustain FOXP3 expression in Tregs and maintains their immunosuppressive function (Liu Y. et al., 2022). Loss of METTL14 reduces Treg numbers and function, diminishing maternal immune tolerance and elevating miscarriage risk (Liu Y. et al., 2022). Additionally, m6A regulatory enzymes such as ALKBH5, YTHDF3, and YTHDC1 are involved in HLA gene expression regulation. Imbalances in their expression can disrupt maternal-fetal immune homeostasis and increase RSA risk (Zhang X. et al., 2021; Sun et al., 2021).

#### 1.4.2 Endocrine disorders

Approximately 20% of RSA cases are associated with endocrine dysfunction (Quenby et al., 2021; Khalife et al., 2019), most commonly involving progesterone deficiency and thyroid dysfunction.

##### 1.4.2.1 Progesterone

Progesterone is central to embryo implantation and early gestation. Reduced progesterone levels are found in many RSA patients (Coomarasamy et al., 2021; Duane et al., 2022). Its action is mediated through the progesterone receptor (PGR). Studies show that PGR mRNA expression is regulated by METTL3-mediated m6A methylation, and METTL3 deficiency significantly reduces PGR levels (Zheng et al., 2023). YTHDF1 is also involved in PGR mRNA translation. Luteal phase insufficiency and low progesterone impair endometrial support, leading to implantation failure and early loss (Khalife et al., 2019). In autoimmune RSA models, exogenous progesterone supplementation improves outcomes by upregulating the Treg/NK cell ratio and reducing pro-inflammatory cytokines (Chen et al., 2021).

##### 1.4.2.2 Thyroid function

Thyroid autoimmunity (TAI), indicated by positivity for thyroid peroxidase antibody (TPO-Ab) and elevated thyroid-stimulating hormone (TSH), is significantly associated with early miscarriage in RSA patients (Wang L. et al., 2021). Polymorphisms in the FTO gene are closely associated with TSH levels, with certain genotypes identified as risk factors for elevated TSH (Wang L. et al., 2021). FTO, highly expressed in the hypothalamus and pituitary, regulates TSH synthesis and secretion by modulating mRNA methylation, influencing the hypothalamic-pituitary-thyroid axis (Dwivedi et al., 2012).

#### 1.4.3 Obesity

Obesity is a significant risk factor for miscarriage, involving oxidative stress, systemic inflammation, reduced uterine receptivity, and impaired oocyte metabolism (Eapen et al., 2021). This risk is heightened in women with a history of RSA (Dimitriadis et al., 2020; Cavalcante et al., 2019). The development of obesity is regulated by methylation mechanisms. The FTO gene is a well-known susceptibility gene for adipogenesis, regulating adipocyte differentiation through m6A demethylation (Frayling et al., 2007; Locke et al., 2015). Its risk allele (e.g., rs9939609 A) increases body mass index (BMI) and indirectly raises the risk of type 2 diabetes and recurrent miscarriage. FTO’s high hypothalamic expression also suggests a role in central energy intake regulation, potentially disrupting ovulation and endometrial preparation (Frayling et al., 2007).

Other m6A-associated enzymes affect lipid synthesis. Increased METTL3 expression can suppress adipocyte proliferation, while METTL3 deficiency reduces m6A on fatty acid synthase (Fasn)mRNA, impairing fatty acid metabolism (Liu et al., 2019; Xie et al., 2019). In hepatic tissue, METTL3 promotes lipid synthesis by stabilizing lipogenic genes; its overexpression exacerbates hepatic fat accumulation and insulin resistance (Xie et al., 2019). In hyperlipidemic mouse livers, METTL3 deficiency reduces global m6A and hepatic lipid accumulation by increasing the stability of metabolic genes like Lpin1 (Li et al., 2020). The reader YTHDC2 is upregulated in hepatocytes in obesity and non-alcoholic fatty liver disease (NAFLD), associated with triglyceride accumulation. YTHDC2 recognizes and degrades m6A-modified lipogenic gene mRNAs, inhibiting excessive lipid synthesis (Zhou et al., 2021; De Jesus et al., 2019).

#### 1.4.4 Thrombosis

Thrombophilia is a major cause of miscarriage, disrupting the balance between coagulation and fibrinolysis essential for placental perfusion. m6A RNA methylation may participate in thrombosis by regulating gene expression. During venous thromboembolism (VTE), activated neutrophils release neutrophil extracellular traps (NETs), which disrupt endothelial barrier function and promote iron-dependent lipid peroxidation (ferroptosis), fostering thrombosis (Li et al., 2024).

NETs markedly increase m6A RNA modification levels in endothelial cells, particularly by upregulating METTL3, which enhances m6A methylation and stability of TLR4 mRNA. This activates the TLR4/NF-κB pathway, promoting pro-inflammatory cytokine release, oxidative stress, and ferroptosis—a vicious cycle of inflammation-ferroptosis-thrombosis. Inhibition of METTL3 can reverse TLR4/NF-κB activation, alleviating endothelial damage and thrombogenesis (Li et al., 2024; Zhang et al., 2024).

Bioinformatics analyses reveal aberrant expression of various m6A regulatory factors (e.g., METTL3, RBM15, FTO, YTHDF1/3, HNRNPC) in the peripheral blood of VTE patients, associated with altered immune cell infiltration and autophagy. Among these, YTHDF3 is a central node linking m6A modification, immune responses, and autophagy, exacerbating inflammation and thrombosis (Zhang et al., 2024).

## 2 Conclusion

RSA poses a significant threat to women’s reproductive health, imposing substantial burdens on patients, families, and society. Its etiology is more complex and less understood than that of sporadic abortion, and treatment options remain limited. Therefore, investigating novel, multifaceted therapeutic strategies targeting its pathogenesis is imperative.

Epigenetics, particularly the reversible m6A RNA methylation which does not alter the genetic code, stands out as a promising field. It offers fresh insights and potential targets for developing diverse therapeutic approaches aimed at addressing the pathogenesis of RSA. In this review, we have examined the biological functions of m6A in key stages of pregnancy establishment and maintenance, and explored its potential involvement in pathways influencing established risk factors for RSA.

## References

[B2] Abdukassimova M. Kanabekova P. Bauyrzhanova Z. Ukybassova T. Kaldygulova L. Imankulova B. (2021). Association of human forkhead box protein 3 (FOXP3) gene polymorphisms with idiopathic recurrent pregnancy loss among kazakhstani women. Gene 801, 145835. 10.1016/j.gene.2021.145835 34274475

[B3] Aik W. Scotti J. S. Choi H. Gong L. Demetriades M. Schofield C. J. (2014). Structure of human RNA N(6)-methyladenine demethylase ALKBH5 provides insights into its mechanisms of nucleic acid recognition and demethylation. Nucleic Acids Res. 42 (7), 4741–4754. 10.1093/nar/gku085 24489119 PMC3985658

[B4] Aimagambetova G. Hajjej A. Malalla Z. H. Finan R. R. Sarray S. Almawi W. Y. (2019). HLA-DQ, and HLA-DP loci are linked with altered risk of recurrent pregnancy loss in Lebanese women: a case-control study. Am. J. Reprod. Immunol. 82 (4), e13173. 10.1111/aji.13173 31339184

[B5] Alarcon C. R. Goodarzi H. Lee H. Liu X. Tavazoie S. Tavazoie S. F. (2015). HNRNPA2B1 is a mediator of m(6)A-Dependent nuclear RNA processing events. Cell. 162 (6), 1299–1308. 10.1016/j.cell.2015.08.011 26321680 PMC4673968

[B6] Alecsandru D. Klimczak A. M. Garcia Velasco J. A. Pirtea P. Franasiak J. M. (2021). Immunologic causes and thrombophilia in recurrent pregnancy loss. Fertil. Steril. 115 (3), 561–566. 10.1016/j.fertnstert.2021.01.017 33610320

[B7] Alijotas-Reig J. Llurba E. Gris J. M. (2014). Potentiating maternal immune tolerance in pregnancy: a new challenging role for regulatory T cells. Placenta 35 (4), 241–248. 10.1016/j.placenta.2014.02.004 24581729

[B8] Aruna M. Nagaraja T. Andal Bhaskar S. Tarakeswari S. Reddy A. G. Thangaraj K. (2011). Novel alleles of HLA-DQ and -DR loci show association with recurrent miscarriages among South Indian women. Hum. Reprod. 26 (4), 765–774. 10.1093/humrep/der024 21325036

[B9] Bahia W. Zitouni H. Kanabekova P. Bauyrzhanova Z. Shaimardanova M. Finan R. R. (2022). Human forkhead box protein 3 gene variants associated with altered susceptibility to idiopathic recurrent pregnancy loss: a retrospective case-control study. Am. J. Reprod. Immunol. 88 (2), e13551. 10.1111/aji.13551 35452532

[B10] Bai L. Xiang Y. Tang M. Liu S. Chen Q. Chen Q. (2023). ALKBH5 controls the meiosis-coupled mRNA clearance in oocytes by removing the N (6)-methyladenosine methylation. Nat. Commun. 14 (1), 6532. 10.1038/s41467-023-42302-6 37848452 PMC10582257

[B11] Bansal A. S. (2010). Joining the immunological dots in recurrent miscarriage. Am. J. Reprod. Immunol. 64 (5), 307–315. 10.1111/j.1600-0897.2010.00864.x 20528832

[B12] Berulava T. Buchholz E. Elerdashvili V. Pena T. Islam M. R. Lbik D. (2020). Changes in m6A RNA methylation contribute to heart failure progression by modulating translation. Eur. J. Heart Fail 22 (1), 54–66. 10.1002/ejhf.1672 31849158

[B13] Bian Y. Li J. Shen H. Li Y. Hou Y. Huang L. (2022). WTAP dysregulation-mediated HMGN3-m6A modification inhibited trophoblast invasion in early-onset preeclampsia. FASEB J. 36 (12), e22617. 10.1096/fj.202200700RR 36412513

[B14] Bokar J. A. Shambaugh M. E. Polayes D. Matera A. G. Rottman F. M. (1997). Purification and cDNA cloning of the AdoMet-binding subunit of the human mRNA (N6-adenosine)-methyltransferase. RNA 3 (11), 1233–1247. 9409616 PMC1369564

[B15] Cao Z. Zhang D. Wang Y. Tong X. Avalos L. F. C. Khan I. M. (2020). Identification and functional annotation of m6A methylation modification in granulosa cells during antral follicle development in pigs. Anim. Reprod. Sci. 219, 106510. 10.1016/j.anireprosci.2020.106510 32828396

[B16] Cavalcante M. B. Sarno M. Peixoto A. B. Araujo Junior E. Barini R. (2019). Obesity and recurrent miscarriage: a systematic review and meta-analysis. J. Obstet. Gynaecol. Res. 45 (1), 30–38. 10.1111/jog.13799 30156037

[B17] Chang Z. Kuang H. X. Zhou X. Zhu H. Zhang Y. Fu Y. (2022). Temporal changes in cyclinD-CDK4/CDK6 and cyclinE-CDK2 pathways: implications for the mechanism of deficient decidualization in an immune-based mouse model of unexplained recurrent spontaneous abortion. Mol. Med. 28 (1), 100. 10.1186/s10020-022-00523-3 36050637 PMC9438304

[B18] Chen Y. Wu Q. Wei J. Hu J. Zheng S. (2021). Effects of aspirin, vitamin D3, and progesterone on pregnancy outcomes in an autoimmune recurrent spontaneous abortion model. Braz J. Med. Biol. Res. 54 (9), e9570. 10.1590/1414-431X2020e9570 34133541 PMC8208775

[B19] Coomarasamy A. Dhillon-Smith R. K. Papadopoulou A. Al-Memar M. Brewin J. Abrahams V. M. (2021). Recurrent miscarriage: evidence to accelerate action. Lancet 397 (10285), 1675–1682. 10.1016/S0140-6736(21)00681-4 33915096

[B20] Cui L. Shi M. Meng X. Qian J. Wang S. (2023). Identification of m6A modification regulated by dysregulated circRNAs in Decidua of recurrent pregnancy loss. Curr. Issues Mol. Biol. 45 (11), 8767–8779. 10.3390/cimb45110551 37998728 PMC10670759

[B21] De Jesus D. F. Zhang Z. Kahraman S. Brown N. K. Chen M. Hu J. (2019). m(6)A mRNA methylation regulates human beta-cell biology in physiological States and in type 2 diabetes. Nat. Metab. 1 (8), 765–774. 10.1038/s42255-019-0089-9 31867565 PMC6924515

[B22] Dimitriadis E. Menkhorst E. Saito S. Kutteh W. H. Brosens J. J. (2020). Recurrent pregnancy loss. Nat. Rev. Dis. Prim. 6 (1), 98. 10.1038/s41572-020-00228-z 33303732

[B23] Dominissini D. Moshitch-Moshkovitz S. Schwartz S. Salmon-Divon M. Ungar L. Osenberg S. (2012). Topology of the human and mouse m6A RNA methylomes revealed by m6A-seq. Nature 485 (7397), 201–206. 10.1038/nature11112 22575960

[B24] Duan M. Liu H. Xu S. Yang Z. Zhang F. Wang G. (2024). IGF2BPs as novel m(6)A readers: diverse roles in regulating cancer cell biological functions, hypoxia adaptation, metabolism, and immunosuppressive tumor microenvironment. Genes Dis. 11 (2), 890–920. 10.1016/j.gendis.2023.06.017 37692485 PMC10491980

[B25] Duane M. Schliep K. Porucznik C. A. Najmabadi S. Stanford J. B. (2022). Does a short luteal phase correlate with an increased risk of miscarriage? A cohort study. BMC Pregnancy Childbirth 22 (1), 922. 10.1186/s12884-022-05195-9 36482355 PMC9733331

[B26] Dwivedi O. P. Tabassum R. Chauhan G. Ghosh S. Marwaha R. K. Tandon N. (2012). Common variants of FTO are associated with childhood obesity in a cross-sectional study of 3,126 urban Indian children. PLoS One 7 (10), e47772. 10.1371/journal.pone.0047772 23091647 PMC3472993

[B27] Eapen A. Hayes E. T. McQueen D. B. Beestrum M. Eyck P. T. Boots C. (2021). Mean differences in maternal body mass index and recurrent pregnancy loss: a systematic review and meta-analysis of observational studies. Fertil. Steril. 116 (5), 1341–1348. 10.1016/j.fertnstert.2021.06.019 34412893 PMC8608000

[B28] Erlebacher A. (2013). Immunology of the maternal-fetal interface. Annu. Rev. Immunol. 31, 387–411. 10.1146/annurev-immunol-032712-100003 23298207

[B29] Fan Z. Wang Q. Deng H. (2021). Circ_0011460 upregulates HTRA1 expression by sponging miR-762 to suppress HTR8/SVneo cell growth, migration, and invasion. Am. J. Reprod. Immunol. 86 (5), e13485. 10.1111/aji.13485 34270834

[B30] Feng C. W. Bowles J. Koopman P. (2014). Control of mammalian germ cell entry into meiosis. Mol. Cell. Endocrinol. 382 (1), 488–497. 10.1016/j.mce.2013.09.026 24076097

[B31] Frayling T. M. Timpson N. J. Weedon M. N. Zeggini E. Freathy R. M. Lindgren C. M. (2007). A common variant in the FTO gene is associated with body mass index and predisposes to childhood and adult obesity. Science 316 (5826), 889–894. 10.1126/science.1141634 17434869 PMC2646098

[B32] Fu Y. Jia G. Pang X. Wang R. N. Wang X. Li C. J. (2013). FTO-mediated formation of N6-hydroxymethyladenosine and N6-formyladenosine in Mammalian RNA. Nat. Commun. 4, 1798. 10.1038/ncomms2822 23653210 PMC3658177

[B33] Gellersen B. Brosens J. J. (2014). Cyclic decidualization of the human endometrium in reproductive health and failure. Endocr. Rev. 35 (6), 851–905. 10.1210/er.2014-1045 25141152

[B34] Gentric G. Kieffer Y. Mieulet V. Goundiam O. Bonneau C. Nemati F. (2019). PML-regulated mitochondrial metabolism enhances chemosensitivity in human ovarian cancers. Cell. Metab. 29 (1), 156–173.e110. 10.1016/j.cmet.2018.09.002 30244973 PMC6331342

[B35] Gilbert W. V. Bell T. A. Schaening C. (2016). Messenger RNA modifications: form, distribution, and function. Science 352 (6292), 1408–1412. 10.1126/science.aad8711 27313037 PMC5094196

[B36] Hsu P. J. Zhu Y. Ma H. Guo Y. Shi X. Liu Y. (2017). Ythdc2 is an N(6)-methyladenosine binding protein that regulates Mammalian spermatogenesis. Cell. Res. 27 (9), 1115–1127. 10.1038/cr.2017.99 28809393 PMC5587856

[B37] Huang E. Chen L. (2023). RNA N(6)-methyladenosine modification in female reproductive biology and pathophysiology. Cell. Commun. Signal 21 (1), 53. 10.1186/s12964-023-01078-4 36894952 PMC9996912

[B38] Huang H. Weng H. Sun W. Qin X. Shi H. Wu H. (2018). Recognition of RNA N(6)-methyladenosine by IGF2BP proteins enhances mRNA stability and translation. Nat. Cell. Biol. 20 (3), 285–295. 10.1038/s41556-018-0045-z 29476152 PMC5826585

[B39] Huang N. Gao Y. Zhang M. Guo L. Qin L. Liao S. (2022a). METTL3-Mediated m(6)A RNA methylation of ZBTB4 interferes with trophoblast invasion and maybe involved in RSA. Front. Cell. Dev. Biol. 10, 894810. 10.3389/fcell.2022.894810 35774226 PMC9237410

[B40] Huang Q. Mo J. Liao Z. Chen X. Zhang B. (2022b). The RNA m(6)A writer WTAP in diseases: structure, roles, and mechanisms. Cell. Death Dis. 13 (10), 852. 10.1038/s41419-022-05268-9 36207306 PMC9546849

[B41] Ivanova I. Much C. Di Giacomo M. Azzi C. Morgan M. Moreira P. N. (2017). The RNA m(6)A reader YTHDF2 is essential for the post-transcriptional regulation of the maternal transcriptome and oocyte competence. Mol. Cell. 67 (6), 1059–1067 e1054. 10.1016/j.molcel.2017.08.003 28867294 PMC5613143

[B42] Jaslow C. R. Carney J. L. Kutteh W. H. (2010). Diagnostic factors identified in 1020 women with two *versus* three or more recurrent pregnancy losses. Fertil. Steril. 93 (4), 1234–1243. 10.1016/j.fertnstert.2009.01.166 19338986

[B43] Jia G. Fu Y. Zhao X. Dai Q. Zheng G. Yang Y. (2011). N6-methyladenosine in nuclear RNA is a major substrate of the obesity-associated FTO. Nat. Chem. Biol. 7 (12), 885–887. 10.1038/nchembio.687 22002720 PMC3218240

[B44] Jiang X. Liu B. Nie Z. Duan L. Xiong Q. Jin Z. (2021). The role of m6A modification in the biological functions and diseases. Signal Transduct. Target Ther. 6 (1), 74. 10.1038/s41392-020-00450-x 33611339 PMC7897327

[B45] Jia J. Wu S. Jia Z. Wang C. Ju C. Sheng J. (2022). Novel insights into m(6)A modification of coding and non-coding RNAs in tumor biology: from molecular mechanisms to therapeutic significance. Int. J. Biol. Sci. 18 (11), 4432–4451. 10.7150/ijbs.73093 35864970 PMC9295064

[B46] Kadyrov M. Kingdom J. C. Huppertz B. (2006). Divergent trophoblast invasion and apoptosis in placental bed spiral arteries from pregnancies complicated by maternal anemia and early-onset preeclampsia/intrauterine growth restriction. Am. J. Obstet. Gynecol. 194 (2), 557–563. 10.1016/j.ajog.2005.07.035 16458661

[B47] Kagawa H. Javali A. Khoei H. H. Sommer T. M. Sestini G. Novatchkova M. (2022). Human blastoids model blastocyst development and implantation. Nature 601 (7894), 600–605. 10.1038/s41586-021-04267-8 34856602 PMC8791832

[B48] Kasowitz S. D. Ma J. Anderson S. J. Leu N. A. Xu Y. Gregory B. D. (2018). Nuclear m6A reader YTHDC1 regulates alternative polyadenylation and splicing during mouse oocyte development. PLoS Genet. 14 (5), e1007412. 10.1371/journal.pgen.1007412 29799838 PMC5991768

[B49] Khalife D. Ghazeeri G. Kutteh W. (2019). Review of current guidelines for recurrent pregnancy loss: new strategies for optimal evaluation of women who May be superfertile. Semin. Perinatol. 43 (2), 105–115. 10.1053/j.semperi.2018.12.008 30642578

[B50] La Rocca C. Carbone F. Longobardi S. Matarese G. (2014). The immunology of pregnancy: regulatory T cells control maternal immune tolerance toward the fetus. Immunol. Lett. 162 (1 Pt A), 41–48. 10.1016/j.imlet.2014.06.013 24996040

[B51] Laisk T. Tsuiko O. Jatsenko T. Horak P. Otala M. Lahdenpera M. (2019). Demographic and evolutionary trends in ovarian function and aging. Hum. Reprod. Update 25 (1), 34–50. 10.1093/humupd/dmy031 30346539

[B52] Lee K. Y. DeMayo F. J. (2004). Animal models of implantation. Reproduction 128 (6), 679–695. 10.1530/rep.1.00340 15579585

[B53] Lee M. Kim B. Kim V. N. (2014). Emerging roles of RNA modification: M(6)A and U-tail. Cell. 158 (5), 980–987. 10.1016/j.cell.2014.08.005 25171402

[B54] Li R. Albertini D. F. (2013). The road to maturation: somatic cell interaction and self-organization of the Mammalian oocyte. Nat. Rev. Mol. Cell. Biol. 14 (3), 141–152. 10.1038/nrm3531 23429793

[B55] Li X. C. Jin F. Wang B. Y. Yin X. J. Hong W. Tian F. J. (2019). The m6A demethylase ALKBH5 controls trophoblast invasion at the maternal-fetal interface by regulating the stability of CYR61 mRNA. Theranostics 9 (13), 3853–3865. 10.7150/thno.31868 31281518 PMC6587351

[B56] Li Y. Zhang Q. Cui G. Zhao F. Tian X. Sun B. F. (2020). m(6)A regulates liver metabolic disorders and hepatogenous diabetes. Genomics Proteomics Bioinforma. 18 (4), 371–383. 10.1016/j.gpb.2020.06.003 33160098 PMC8242261

[B57] Li X. Jin J. Long X. Weng R. Xiong W. Liang J. (2023). METTL3-regulated m6A modification impairs the decidualization of endometrial stromal cells by regulating YTHDF2-mediated degradation of FOXO1 mRNA in endometriosis-related infertility. Reprod. Biol. Endocrinol. 21 (1), 99. 10.1186/s12958-023-01151-0 37891533 PMC10605339

[B58] Li Y. Gu J. Ge J. Kong J. Shang L. (2024). HSYA ameliorates venous thromboembolism by depleting the formation of TLR4/NF-κB pathway-dependent neutrophil extracellular traps. Int. Immunopharmacol. 143 (Pt 3), 113534. 10.1016/j.intimp.2024.113534 39504860

[B59] Lin Z. Hsu P. J. Xing X. Fang J. Lu Z. Zou Q. (2017). Mettl3-/Mettl14-mediated mRNA N(6)-methyladenosine modulates murine spermatogenesis. Cell. Res. 27 (10), 1216–1230. 10.1038/cr.2017.117 28914256 PMC5630681

[B60] Lin X. Dai Y. Gu W. Zhang Y. Zhuo F. Zhao F. (2024). The involvement of RNA N6-methyladenosine and histone methylation modification in decidualization and endometriosis-associated infertility. Clin. Transl. Med. 14 (2), e1564. 10.1002/ctm2.1564 38344897 PMC10859880

[B61] Liu N. Dai Q. Zheng G. He C. Parisien M. Pan T. (2015). N(6)-methyladenosine-dependent RNA structural switches regulate RNA-Protein interactions. Nature 518 (7540), 560–564. 10.1038/nature14234 25719671 PMC4355918

[B62] Liu Q. Zhao Y. Wu R. Jiang Q. Cai M. Bi Z. (2019). ZFP217 regulates adipogenesis by controlling mitotic clonal expansion in a METTL3-m(6)A dependent manner. RNA Biol. 16 (12), 1785–1793. 10.1080/15476286.2019.1658508 31434544 PMC7567449

[B63] Liu H. T. Zou Y. X. Zhu W. J. Sen L. Zhang G. H. Ma R. R. (2022a). lncRNA THAP7-AS1, transcriptionally activated by SP1 and post-transcriptionally stabilized by METTL3-mediated m6A modification, exerts oncogenic properties by improving CUL4B entry into the nucleus. Cell. Death Differ. 29 (3), 627–641. 10.1038/s41418-021-00879-9 34608273 PMC8901790

[B64] Liu Y. Yuan Y. Zhou Z. Cui Y. Teng Y. Huang H. (2022b). Mettl14-mediated m6A modification enhances the function of Foxp3(+) regulatory T cells and promotes allograft acceptance. Front. Immunol. 13, 1022015. 10.3389/fimmu.2022.1022015 36341394 PMC9629694

[B65] Liu B. Liu J. Qiu Y. Chen J. Yang J. (2023). MITA promotes macrophage proinflammatory polarization and its circRNA-Related regulatory mechanism in recurrent miscarriage. Int. J. Mol. Sci. 24 (11), 9545. 10.3390/ijms24119545 37298501 PMC10253871

[B66] Locke A. E. Kahali B. Berndt S. I. Justice A. E. Pers T. H. Day F. R. (2015). Genetic studies of body mass index yield new insights for obesity biology. Nature 518 (7538), 197–206. 10.1038/nature14177 25673413 PMC4382211

[B67] Lucas E. S. Vrljicak P. Muter J. Diniz-da-Costa M. M. Brighton P. J. Kong C. S. (2020). Recurrent pregnancy loss is associated with a pro-senescent decidual response during the peri-implantation window. Commun. Biol. 3 (1), 37. 10.1038/s42003-020-0763-1 31965050 PMC6972755

[B68] Meuleman T. Lashley L. E. Dekkers O. M. van Lith J. M. Claas F. H. Bloemenkamp K. W. (2015). HLA associations and HLA sharing in recurrent miscarriage: a systematic review and meta-analysis. Hum. Immunol. 76 (5), 362–373. 10.1016/j.humimm.2015.02.004 25700963

[B69] Meuleman T. Haasnoot G. W. van Lith J. M. M. Verduijn W. Bloemenkamp K. W. M. Claas F. H. J. (2018). Paternal HLA-C is a risk factor in unexplained recurrent miscarriage. Am. J. Reprod. Immunol. 79 (2), e12797. 10.1111/aji.12797 29205643

[B70] Ng S. W. Norwitz G. A. Pavlicev M. Tilburgs T. Simon C. Norwitz E. R. (2020). The primary driver of pregnancy health. Int. J. Mol. Sci. 21 (11). 10.3390/ijms21114092 PMC7312091 32521725

[B71] Owusu-Akyaw A. Krishnamoorthy K. Goldsmith L. T. Morelli S. S. (2019). The role of mesenchymal-epithelial transition in endometrial function. Hum. Reprod. Update 25 (1), 114–133. 10.1093/humupd/dmy035 30407544

[B72] Ping X. L. Sun B. F. Wang L. Xiao W. Yang X. Wang W. J. (2014). Mammalian WTAP is a regulatory subunit of the RNA N6-methyladenosine methyltransferase. Cell. Res. 24 (2), 177–189. 10.1038/cr.2014.3 24407421 PMC3915904

[B73] Practice Committee of the American Society for Reproductive (2012). Evaluation and treatment of recurrent pregnancy loss: a committee opinion. Fertil. Steril. 98 (5), 1103–1111. 10.1016/j.fertnstert.2012.06.048 22835448

[B74] Prins J. R. Kieffer T. E. Scherjon S. A. (2014). Immunomodulators to treat recurrent miscarriage. Eur. J. Obstet. Gynecol. Reprod. Biol. 181, 334–337. 10.1016/j.ejogrb.2014.07.038 25156972

[B75] Qi Z. Liu Y. Yang H. Yang X. Wang H. Liu B. (2022). Protective role of m(6)A binding protein YTHDC2 on CCNB2 in manganese-induced spermatogenesis dysfunction. Chem. Biol. Interact. 351, 109754. 10.1016/j.cbi.2021.109754 34822792

[B76] Qiu W. Zhou Y. Wu H. Lv X. Yang L. Ren Z. (2021). RNA demethylase FTO mediated RNA m(6)A modification is involved in maintaining maternal-fetal interface in spontaneous abortion. Front. Cell. Dev. Biol. 9, 617172. 10.3389/fcell.2021.617172 34350169 PMC8326377

[B77] Quenby S. Gallos I. D. Dhillon-Smith R. K. Podesek M. Stephenson M. D. Fisher J. (2021). Miscarriage matters: the epidemiological, physical, psychological, and economic costs of early pregnancy loss. Lancet 397 (10285), 1658–1667. 10.1016/S0140-6736(21)00682-6 33915094

[B78] Rai R. Regan L. (2006). Recurrent miscarriage. Lancet 368 (9535), 601–611. 10.1016/S0140-6736(06)69204-0 16905025

[B1] Recurrent abortion (1993). Recurrent abortion. J. Reprod. Med. 38 (4), 250–259. 8501731

[B79] Roundtree I. A. Evans M. E. Pan T. He C. (2017). Dynamic RNA modifications in gene expression regulation. Cell. 169 (7), 1187–1200. 10.1016/j.cell.2017.05.045 28622506 PMC5657247

[B80] Salker M. Teklenburg G. Molokhia M. Lavery S. Trew G. Aojanepong T. (2010). Natural selection of human embryos: impaired decidualization of endometrium disables embryo-maternal interactions and causes recurrent pregnancy loss. PLoS One 5 (4), e10287. 10.1371/journal.pone.0010287 20422017 PMC2858209

[B81] Sendinc E. Shi Y. (2023). RNA m6A methylation across the transcriptome. Mol. Cell. 83 (3), 428–441. 10.1016/j.molcel.2023.01.006 36736310

[B82] Shi H. Wang X. Lu Z. Zhao B. S. Ma H. Hsu P. J. (2017). YTHDF3 facilitates translation and decay of N(6)-methyladenosine-modified RNA. Cell. Res. 27 (3), 315–328. 10.1038/cr.2017.15 28106072 PMC5339834

[B83] Shi H. Wei J. He C. (2019). Where, when, and how: context-dependent functions of RNA methylation writers, readers, and erasers. Mol. Cell. 74 (4), 640–650. 10.1016/j.molcel.2019.04.025 31100245 PMC6527355

[B84] Sun D. Yang H. Fan L. Shen F. Wang Z. (2021). m6A regulator-mediated RNA methylation modification patterns and immune microenvironment infiltration characterization in severe asthma. J. Cell. Mol. Med. 25 (21), 10236–10247. 10.1111/jcmm.16961 34647423 PMC8572790

[B85] Tang C. Klukovich R. Peng H. Wang Z. Yu T. Zhang Y. (2018). ALKBH5-dependent m6A demethylation controls splicing and stability of long 3'-UTR mRNAs in Male germ cells. Proc. Natl. Acad. Sci. U. S. A. 115 (2), E325–E333. 10.1073/pnas.1717794115 29279410 PMC5777073

[B86] Tsui M. H. Pang M. W. Melender H. L. Xu L. Lau T. K. Leung T. N. (2006). Maternal fear associated with pregnancy and childbirth in Hong Kong Chinese women. Women Health 44 (4), 79–92. 10.1300/j013v44n04_05 17456465

[B87] Turesheva A. Aimagambetova G. Ukybassova T. Marat A. Kanabekova P. Kaldygulova L. (2023). Recurrent pregnancy loss etiology, risk factors, diagnosis, and management. Fresh look into a full box. J. Clin. Med. 12 (12), 4074. 10.3390/jcm12124074 37373766 PMC10298962

[B88] Vomstein K. Feil K. Strobel L. Aulitzky A. Hofer-Tollinger S. Kuon R. J. (2021). Immunological risk factors in recurrent pregnancy loss: guidelines *versus* current state of the art. J. Clin. Med. 10 (4), 869. 10.3390/jcm10040869 33672505 PMC7923780

[B89] Wang J. Y. Lu A. Q. (2021). The biological function of m6A reader YTHDF2 and its role in human disease. Cancer Cell. Int. 21 (1), 109. 10.1186/s12935-021-01807-0 33593354 PMC7885220

[B90] Wang X. Feng J. Xue Y. Guan Z. Zhang D. Liu Z. (2016). Structural basis of N(6)-adenosine methylation by the METTL3-METTL14 complex. Nature 534 (7608), 575–578. 10.1038/nature18298 27281194

[B91] Wang T. Kong S. Tao M. Ju S. (2020). The potential role of RNA N6-methyladenosine in cancer progression. Mol. Cancer 19 (1), 88. 10.1186/s12943-020-01204-7 32398132 PMC7216508

[B92] Wang Y. Zhang L. Ren H. Ma L. Guo J. Mao D. (2021a). Role of hakai in m(6)A modification pathway in drosophila. Nat. Commun. 12 (1), 2159. 10.1038/s41467-021-22424-5 33846330 PMC8041851

[B93] Wang L. Yi Q. Yao H. He L. Fang B. Xu W. (2021b). Correlations between FTO gene polymorphisms and TSH level in Uyghur Chinese patients with type 2 diabetes. Biomed. Res. Int. 2021, 6646750. 10.1155/2021/6646750 34258276 PMC8257352

[B94] Wang Y. Li Y. Skuland T. Zhou C. Li A. Hashim A. (2023). The RNA m(6)A landscape of mouse oocytes and preimplantation embryos. Nat. Struct. Mol. Biol. 30 (5), 703–709. 10.1038/s41594-023-00969-x 37081317 PMC10337017

[B95] Wang T. Zhang L. Gao W. Liu Y. Yue F. Ma X. (2024). Transcriptome-wide N6-methyladenosine modification profiling of long non-coding RNAs in patients with recurrent implantation failure. BMC Med. Genomics 17 (1), 251. 10.1186/s12920-024-02013-3 39394578 PMC11470675

[B96] Wei J. Yu X. Yang L. Liu X. Gao B. Huang B. (2022). FTO mediates LINE1 m(6)A demethylation and chromatin regulation in mESCs and mouse development. Science 376 (6596), 968–973. 10.1126/science.abe9582 35511947 PMC9746489

[B97] Willyard C. (2017). An epigenetics gold rush: new controls for gene expression. Nature 542 (7642), 406–408. 10.1038/542406a 28230146

[B98] Wu S. Xie H. Su Y. Jia X. Mi Y. Jia Y. (2023). The landscape of implantation and placentation: deciphering the function of dynamic RNA methylation at the maternal-fetal interface. Front. Endocrinol. (Lausanne) 14, 1205408. 10.3389/fendo.2023.1205408 37720526 PMC10499623

[B99] Xiao W. Adhikari S. Dahal U. Chen Y. S. Hao Y. J. Sun B. F. (2016). Nuclear m(6)A reader YTHDC1 regulates mRNA splicing. Mol. Cell. 61 (4), 507–519. 10.1016/j.molcel.2016.01.012 26876937

[B100] Xie Y. Hou W. Song X. Yu Y. Huang J. Sun X. (2016). Ferroptosis: process and function. Cell. Death Differ. 23 (3), 369–379. 10.1038/cdd.2015.158 26794443 PMC5072448

[B101] Xie W. Ma L. L. Xu Y. Q. Wang B. H. Li S. M. (2019). METTL3 inhibits hepatic insulin sensitivity *via* N6-methyladenosine modification of fasn mRNA and promoting fatty acid metabolism. Biochem. Biophys. Res. Commun. 518 (1), 120–126. 10.1016/j.bbrc.2019.08.018 31405565

[B102] Xiong J. He J. Zhu J. Pan J. Liao W. Ye H. (2022). Lactylation-driven METTL3-mediated RNA m(6)A modification promotes immunosuppression of tumor-infiltrating myeloid cells. Mol. Cell. 82 (9), 1660–1677 e1610. 10.1016/j.molcel.2022.02.033 35320754

[B103] Xu L. Li Y. Sang Y. Li D. J. Du M. (2021). Crosstalk between trophoblasts and decidual immune cells: the cornerstone of maternal-fetal immunotolerance. Front. Immunol. 12, 642392. 10.3389/fimmu.2021.642392 33717198 PMC7947923

[B104] Xue P. Zhou W. Fan W. Jiang J. Kong C. Zhou W. (2021). Increased METTL3-mediated m(6)A methylation inhibits embryo implantation by repressing HOXA10 expression in recurrent implantation failure. Reprod. Biol. Endocrinol. 19 (1), 187. 10.1186/s12958-021-00872-4 34906165 PMC8670269

[B105] Yang W. S. Stockwell B. R. (2016). Ferroptosis: death by lipid peroxidation. Trends Cell. Biol. 26 (3), 165–176. 10.1016/j.tcb.2015.10.014 26653790 PMC4764384

[B106] Yao P. Hu G. Niu H. (2022). Hsa_circ_0074371 regulates proliferation, apoptosis, migration, and invasion *via* the miR-582-3p/LRP6 axis in trophoblast cells. Biochem. Genet. 60 (1), 267–285. 10.1007/s10528-021-10095-2 34184135

[B107] Zenclussen A. C. Schumacher A. Zenclussen M. L. Wafula P. Volk H. D. (2007). Immunology of pregnancy: cellular mechanisms allowing fetal survival within the maternal uterus. Expert Rev. Mol. Med. 9 (10), 1–14. 10.1017/S1462399407000294 17462112

[B108] Zhang Z. Theler D. Kaminska K. H. Hiller M. de la Grange P. Pudimat R. (2010). The YTH domain is a novel RNA binding domain. J. Biol. Chem. 285 (19), 14701–14710. 10.1074/jbc.M110.104711 20167602 PMC2863249

[B109] Zhang L. Wan Y. Zhang Z. Jiang Y. Gu Z. Ma X. (2021a). IGF2BP1 overexpression stabilizes PEG10 mRNA in an m6A-dependent manner and promotes endometrial cancer progression. Theranostics 11 (3), 1100–1114. 10.7150/thno.49345 33391523 PMC7738899

[B110] Zhang X. Zhang S. Yan X. Shan Y. Liu L. Zhou J. (2021b). m6A regulator-mediated RNA methylation modification patterns are involved in immune microenvironment regulation of periodontitis. J. Cell. Mol. Med. 25 (7), 3634–3645. 10.1111/jcmm.16469 33724691 PMC8034465

[B111] Zhang D. Fu W. Zhu S. Pan Y. Li R. (2024). RNA methylation patterns, immune characteristics, and autophagy-related mechanisms mediated by N6-methyladenosine (m6A) regulatory factors in venous thromboembolism. BMC Genomics 25 (1), 403. 10.1186/s12864-024-10294-2 38658847 PMC11044431

[B112] Zhao L. Y. Song J. Liu Y. Song C. X. Yi C. (2020). Mapping the epigenetic modifications of DNA and RNA. Protein Cell. 11 (11), 792–808. 10.1007/s13238-020-00733-7 32440736 PMC7647981

[B113] Zhao X. Tian G. G. Fang Q. Pei X. Wang Z. Wu J. (2021). Comparison of RNA m(6)A and DNA methylation profiles between mouse female germline stem cells and STO cells. Mol. Ther. Nucleic Acids 23, 431–439. 10.1016/j.omtn.2020.11.020 33473328 PMC7803632

[B114] Zhao Y. Sun J. Jin L. (2022). The N6-Methyladenosine regulator ALKBH5 mediated stromal cell-macrophage interaction *via* VEGF signaling to promote recurrent spontaneous abortion: a bioinformatic and *in vitro* study. Int. J. Mol. Sci. 23 (24), 15819. 10.3390/ijms232415819 36555463 PMC9785252

[B115] Zhao S. Dong Y. Li Y. Wang Z. Chen Y. Dong Y. (2024). Melatonin alleviates lipopolysaccharide-induced abnormal pregnancy through MTNR1B regulation of m6A. Int. J. Mol. Sci. 25 (2), 733. 10.3390/ijms25020733 38255808 PMC10815701

[B116] Zheng Z. H. Zhang G. L. Jiang R. F. Hong Y. Q. Zhang Q. Y. He J. P. (2023). METTL3 is essential for normal progesterone signaling during embryo implantation *via* m(6)A-mediated translation control of progesterone receptor. Proc. Natl. Acad. Sci. U. S. A. 120 (5), e2214684120. 10.1073/pnas.2214684120 36693099 PMC9945998

[B117] Zhou W. Wang H. Wu X. Long W. Zheng F. Kong J. (2018). The profile analysis of circular RNAs in human placenta of preeclampsia. Exp. Biol. Med. (Maywood) 243 (14), 1109–1117. 10.1177/1535370218813525 30458645 PMC6327373

[B118] Zhou K. I. Shi H. Lyu R. Wylder A. C. Matuszek Z. Pan J. N. (2019). Regulation of Co-transcriptional Pre-mRNA splicing by m(6)A through the low-complexity protein hnRNPG. Mol. Cell. 76 (1), 70–81 e79. 10.1016/j.molcel.2019.07.005 31445886 PMC6778029

[B119] Zhou B. Liu C. Xu L. Yuan Y. Zhao J. Zhao W. (2021). N(6) -Methyladenosine reader protein YT521-B homology domain-containing 2 suppresses liver steatosis by regulation of mRNA stability of lipogenic genes. Hepatology 73 (1), 91–103. 10.1002/hep.31220 32150756

[B120] Zou H. Mao Q. (2022). Circ_0037078 promotes trophoblast cell proliferation, migration, invasion and angiogenesis by miR-576-5p/IL1RAP axis. Am. J. Reprod. Immunol. 87 (1), e13507. 10.1111/aji.13507 34724268

